# Low-molecular-weight-heparin increases Th1- and Th17-associated chemokine levels during pregnancy in women with unexplained recurrent pregnancy loss: a randomised controlled trial

**DOI:** 10.1038/s41598-019-48799-6

**Published:** 2019-08-23

**Authors:** E. Rasmark Roepke, V. Bruno, E. Nedstrand, R. Boij, C. Petersson Strid, E. Piccione, G. Berg, J. Svensson-Arvelund, M. C. Jenmalm, M. Rubér, J. Ernerudh

**Affiliations:** 1 Department of Obstetrics and Gynecology, Skåne University Hospital, Malmö and Lund University, Lund, Sweden; 2 Department of Clinical and Experimental Medicine, Linköping University, Linköping, Sweden; 3 Departmen of Obstetrics and Gynecology, Kalmar Hospital, Kalmar, Sweden; 4 Section of Gynecology and Obstetrics, Academic Department of Biomedicine and Prevention, and Clinical Department of Surgery, Tor Vergata University Hospital, Rome, Italy; 5 Department of Clinical Immunology and Transfusion Medicine, and Department of Clinical and Experimental Medicine, Linköping University, Linköping, Sweden

**Keywords:** Randomized controlled trials, Predictive markers

## Abstract

Low-molecular-weight heparin (LMWH) is widely used to treat recurrent pregnancy loss (RPL) because of its anti-coagulant effects. Although *in vitro* studies have suggested additional immunological effects, these are debated. We therefore investigated whether LMWH could modulate immune responses *in vivo* during pregnancy of women with unexplained RPL. A Swedish open multi-centre randomised controlled trial included 45 women treated with tinzaparin and 42 untreated women. Longitudinally collected plasma samples were obtained at gestational weeks (gw) 6, 18, 28 and 34 and analysed by multiplex bead technology for levels of 11 cytokines and chemokines, chosen to represent inflammation and T-helper subset-associated immunity. Mixed linear models test on LMWH-treated and untreated women showed differences during pregnancy of the Th1-associated chemokines CXCL10 (p = 0.01), CXCL11 (p < 0.001) and the Th17-associated chemokine CCL20 (p = 0.04), while CCL2, CCL17, CCL22, CXCL1, CXCL8, CXCL12, CXCL13 and IL-6 did not differ. Subsequent Student’s t-test showed significantly higher plasma levels of CXCL10 and CXCL11 in treated than untreated women at gw 28 and 34. The consistent increase in the two Th1-associated chemokines suggests a potential proinflammatory and unfavourable effect of LMWH treatment during later stages of pregnancy, when Th1 immunity is known to disrupt immunological tolerance.

## Introduction

For treatment of recurrent pregnancy loss (RPL), low-molecular-weight heparin (LMWH) is used in clinical practice despite the European Society of Human Reproduction and Embryology (ESHRE) guidelines which do not recommend the use of LMWH treatment for women with unexplained RPL (uRPL), because of evidence that treatment does not increase the live birth rate^1,2^. Differences in study design, definition of RPL, randomisation, screening and treatment procedures make it difficult to present strong evidence-based recommendations in systematic reviews and meta analyses of this treatment^1,3–11^.

LMWH is used to treat uRPL based on the rationale that, additional to well-known anticoagulant effects, LMWH may also be involved in regulating processes at the foetal–maternal interface^5,12–14^. Furthermore, it has been hypothesised that LMWH can potentially induce immune modulatory actions^15–22^, which could balance the proinflammatory response implicated in the pathogenesis of RPL^23^. However, we recently reported that LMWH had proinflammatory actions *in vitro*^24^.

During pregnancy, the maternal immune system needs to be modulated in order to harbour the semi-allogeneic and rapidly growing foetus. This modulation is orchestrated by signalling molecules like cytokines and chemokines^25^. Chemokines recruit specific cell types including different subsets of T-helper (Th) cells involved in different types of immune responses, such as Th1-, Th2- and Th17-associated immunity^26–31^. Moreover, in contrast to most cytokines, chemokines are often present in plasma at measurable levels^32^ and can therefore be used as markers of different types of immune responses^26,27^.

The IFN-γ induced chemokines CXCL10 and CXCL11 bind CXCR3, and thereby play an important role in attracting Th1 cells, preferentially expressing this receptor^28,29,31,33,34^. The chemokines CCL17 and CCL22, induced by IL-4 and IL-13, recruit Th2 cells by binding to CCR4, preferentially expressed on this Th subset^28,29,31,35^, while CCL20 and CXCL1, induced by IL-17, recruit Th17 cells, preferentially expressing CCR6, the receptor for CCL20^17,28–31^. CXCL12 is a multipotent T cell and a monocyte-recruiting chemokine which is suggested to promote Th2 immunity in pregnancy^36^. CXCL13 is a potent B-cell attracting chemokine^28^, while CXCL8 is a potent recruiter of neutrophil granulocytes^37^. CCL2 recruits and activates Th2 cells and has a role in M2-associated foetal tolerance in pregnancy and in reducing macrophage production of proinflammatory cytokines^37,38^. In brief, a successful pregnancy is characterised by increases in Th2 and regulatory T (Treg) cell associated immunity, while Th1 and probably also Th17 associated immunity are unfavourable^39–42^ Notably, uRPL was recently reported to be associated with an increase in Th1 and Th17 immunity^43^.

Little is known about how LMWH affects the immune response *in vivo* during pregnancy. The present study therefore assessed the immunological effects of LMWH treatment during pregnancy in women with uRPL, by longitudinally measuring plasma levels of cytokines and chemokines during the course of pregnancy in LMWH-treated and untreated women participating in a randomised controlled trial with the main outcome measure being immune modulatory effects of LMWH.

## Materials and Methods

An open multi-centre randomised controlled trial, registered at EudraCT (protocol number: 2010-022715-19) was conducted in Sweden from 1 January 2012 to 31 December 2015, where pregnant women with a history of uRPL were included from six centres in the south of Sweden: Helsingborg, Lund, Kalmar, Jönköping, Karlskrona and Linköping. The primary outcome measure was immune modulation throughout pregnancy. Women with uRPL who were referred to one of the gynaecological clinics or a midwife centre were invited to participate in the study.

### Eligible participants

Women who presented to the above units with uRPL, defined as three or more spontaneous consecutive miscarriages before 22 weeks of gestation without a known cause of RPL, were eligible for participation in the study. Only pregnant women were included and pregnancy was determined after confirming viable pregnancy by ultrasound. Inclusion and exclusion criteria (shown in supplementary Table I) were applied to those who accepted participation in the study, based on medical history and records. Participating women had previously undergone a standardised diagnostic workup according to national recommendations, including: a) collection of familial and personal medical, gynecological and obstetrical history with specific references to previous miscarriages; b) gynecological examination; c) transvaginal ultrasound; d) hysterosonography; e) endocrine evaluation panel: TSH, FT4; e) karyotype of both partners; f) immunity panel: anti-phospholipid antibodies, lupus anticoagulant, anti-cardiolipin antibodies, anti-β2GPI, anti-thyroid antibodies (anti-thyroid peroxidase and thyroid receptor antibodies); g) thrombophilia screening: protein C, protein S, homocysteine and determination of the following mutations: factor V Leiden, factor II prothrombin. This workup was aimed to identify proven causes of RPL. When all the above known causes for RPL had been excluded, women were diagnosed with uRPL and were included in this study. Written informed consent was obtained from all participants.

**Figure 1:**
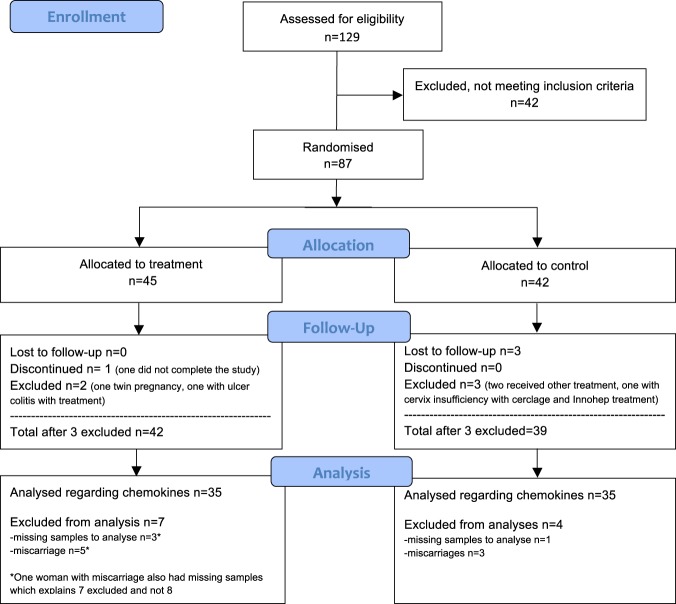
Flow chart of study participants from recruitment to analysis of chemokines.

From the 129 women who were assessed for eligibility, 87 were included for randomisation: 45 women to treatment and 42 women to a control group. Charactaristics of included women is presented in Table 1. Another six patients were excluded during the study because of lack of compliance to the study protocol or fulfilling withdrawal criteria (Fig. 1, Supplementary Table 1), resulting in 81 patients completing the study to the end of their pregnancies. Eight women miscarried and 73 continued beyond 22 gestational weeks (gw), see Table 2. One woman with miscarriage and another three women with full-term live births had missing blood samples, and were therefore excluded from the analyses regarding cytokines and chemokines.

**Table 1:** Characteristics of included women.

Characteristic mean (+−SD) or n	LMWH n = 45	Control n = 42	p-value
Age	33.0 (4.9)	32.8 (4.6)	0.89
BMI	24.8 (4.2)	24.0 (4.0)	0.29
Gravidity	5.1 (2.0)	4.4 (1.3)	0.18
Previous miscarriages	4.0 (1.6)	3.6 (0.9)	0.25
Primary RPL^**a**^	20	23	0.34
Previous live births	0.8 (0.9)	0.6 (0.9)	0.38
**1 child**	18	15	
**2 children**	6	3	
**3 children**	1	1	
Gestational week at enrolment	6.6 (1.3)	6.9 (1.6)	0.52

LMWH; low-molecular-weight heparin, RPL; recurent pregnancy loss, BMI; Body Mass Index kg/m^2^.

^a^Primary RPL when there were no live births before three consecutive miscarriages.

**Table 2:** Pregnancy outcome.

Pregnancy outcome	LMWH n = 42 n (%)	Control n = 39 n (%)	p-value
**Live birth rate**	36 (86)	35 (90)	1.00
**Miscarriage**	5 (12)	3 (7.7)	1.00
**Full-term birth** > 37 gw	32 (76)	31 (80)	0.88
**Mode of delivery**			
Vaginal delivery	21 (50)	27 (69)	0.16
Caesarean, elective/acute	11 (26), 5/6	6 (15), 1/5	0.19
Vacuum or forceps	5 (12)	3 (7.7)	0.71
**Obstetric complications**			
Preterm birth < 37 gw	5^a^ (12)	5^a^ (13)	1.00
Preeclampsia^b^	2 (4.8)	1 (2.6)	1.00
SGA^b^	3 (7.1)	0	0.24
Birthweight^d^ (SD)	3573 (922)	3502 (521)	0.87
Gestational diabetes	0	1 (2.6)	0.49
Bleeding post-partum > 1 L	0	2 (5.1)	0.23
Placenta abruption	1 (2.4)	0	1.00
IUFD	1 (2.4)	1 (2.6)	1.00
Foetal death	0	1^c^ (2.6)	1.00

LMWH; low-molecular-weight heparin, gw; gestational week, SGA; small for gestational age, SD; standard deviation, IUFD: intrauterine foetal death.

^a^Includes IUFD.

^b^Women with preeclampsia and SGA are not the same women. ^c^Included in live birth rate.

^d^birthweight in grams, mean.

In four women no blood samples were available (LMWH group: 1 miscarriage (gw8), 1 normal childbirth, 1 elective caesarean section. Control group: 1 acute caesarean section), and they could therefore not be analysed for cytokine and chemokine changes during pregnancy.

### Intervention

Once pregnancy was verified by ultrasound, all 87 eligible participants were recruited and randomised to one of the following groups.

*Group 1* (study [LMWH] group) included 45 patients who received tinzaparin sodium (Innohep 4500 IU; LEO Pharma A/S, Denmark) by subcutaneous daily injections until gw 37.

*Group 2* (control group) included 42 patients who neither received active treatment, nor placebo.

### Randomisation

Enrolled patients were randomised through sequentially numbered closed envelopes opened consecutively at each inclusion point. Eligible patients were randomly assigned to one of the two study groups according to the envelope noting the intervention type. Once allocation was obtained, it could not be changed.

### Outcomes

The main outcome measures referred to the potential immunological effects of LMWH *in vivo*. Clinical outcomes are shown as descriptive data including miscarriage rate, occurrence of pregnancy complications and any side effects of the drug used, either to the mothers or their infants.

### Follow up schedule

All study participants followed the national guidelines regarding antenatal care with midwife check-ups during pregnancy and 8 weeks’ postpartum, routine ultrasound screening at gw 18–20 and sometimes an additional chromosome (13, 18, 21) screening with combined ultrasound and blood sampling (PAPP-A, hCG). Additionally, participants underwent a transvaginal ultrasound at gw 10 and 12. Three other scans were done transabdominally around gw 18–20 (routine ultrasound according to national guidelines), 28 and 34 weeks to assess growth estimation, umbilical doppler measurement, cardiotocography (CTG) and questioning for adverse events at all check-ups. The antenatal records of the patients, including foetal viability, drug treatment side effects or any other complications, were recorded on the data collection sheet at each visit up to 2 weeks’ *post partum* or miscarriage. In the LMWH-treated group hemoglobin, thrombocytes, APTt and PK-INR were controlled 2–4 weeks after starting LMWH following the local clinical routines. If no abnormal values in the above parameters were observed, no further hematological check-ups were done.

### Plasma samples collection

Longitudinally collected blood samples were obtained from 87 women with uRPL enrolled in the current study assessing immunological effects of LMWH treatment. Samples were collected at four different time points during pregnancy: inclusion time point, before starting treatment (median gw 6, range 5–10), gw 18 (median 18, range 16–18), gw 28 (median 28, range 26–30), and gw 34 (median 34, range 32–37), and at 2 weeks’ *post partum*. If a woman miscarried, the pregnancy stopped before the second sample time-point, and only inclusion samples were obtained. As we performed paired data analyses to evaluate LMWH effects during pregnancy, women with miscarriages were not included in the further analyses.

Blood samples were collected in BD Vacutainer tubes, and after centrifugation at 1500 g, plasma was aliquoted and stored at −70 °C until use.

### Measurements of cytokines and chemokines with multiplex bead assay

To assess the immunological effects of LMWH *in vivo*, multiplex bead assay kits were used, according to the manufacturer’s protocols (Millipore, Merck KGaA, Darmstadt, Germany), to analyse plasma samples for the following analyses (detection limits are shown in brackets): CCL2 (16 pg/ml), CCL17 (1.0 pg/ml), CCL20 (9.8 pg/ml), CCL22 (16 pg/ml), CXCL1 (16 pg/ml), CXCL8 (1.6 pg/ml), CXCL10 (16 pg/ml), CXCL11 (7.8 pg/ml), CXCL12 (3.9 pg/ml), CXCL13 (3.9 pg/ml), IL-6 (1.6 pg/ml). The analyses were chosen to represent inflammation and immunity associated with subsets of T helper (Th) cells: Th1 (CXL10, CXCL11), Th2 (CCL17, CCL22), Th17 (CCL20, CXCL1, CXCL8), as well as B cells (CXCL13), general inflammation (IL-6, CXCL8), monocyte recruitment and Th2/anti-inflammatory (CCL2) and monocyte/T cell recruitment and Th2 (CXCL12). The measurements were performed using the Luminex 200 IS system (Millipore) and the MasterPlex QT 2010 software (MiraiBio). Values below the detection limit were assigned half the value of the detection limit.

### Data analysis and statistics

A priori analysis showed that the sample size of 70 women was sufficient to detect a 40% decrease or increase in an immunological parameter (CXCL10) with a power of 80% and an *alpha* value of 0.05. Since the Kolmogorov-Smirnov test showed that data on cytokines and chemokines were not normally distributed, they were normalised by logarithmic transformation. First, Linear Mixed Models were used to evaluate differences between treated and untreated women in any of the cytokines and chemokines during the course of pregnancy. If p < 0.05, a post-hoc test was done to decide at which time point(s) and in which direction there was a difference. As only three chemokines were <0.05 in the linear mixed model test, the post-hoc testing in these cases was done with ordinary Student´s t-test or Fisher’s exact tests; the latter was used in one case (CCL20) because of the low proportion of samples with detectable levels. Student´s t-test on log-transformed values was used to analyse differences in cytokine/chemokine levels at inclusion and at 2 weeks’ *post partum*. Data were expressed as geometric mean and 95% confidence intervals in the figures and tables. P values < 0.05 were considered statistically significant. All data were analysed using SPSS version 24 (Armonk, NY: IMB Corp.) and graphs were made using GraphPad Prism version 7.0 (La Jolla, CA, USA).

### Ethical approval

The regional ethical board in Linköping, Sweden, approved the study (*Dnr 2010/295-31*). The study was also approved by the Swedish Medical Products Agency (EudraCT protocol number: 2010-022715-19). All experiments were performed in accordance with the Helsinki Declaration ethical principles for medical research.

### Trial registration number

Eudra-CT number 2010-022715-19. Register date 20/12/2010.

## Results

### Demographic, clinical and immunological data at inclusion

A total of 87 women participated: 45 in the treatment and 42 in the control group (Fig. 1). The two groups were similar in age, parity, number of previous miscarriages, gestational age, and body mass index at the time of enrolment (descriptive data are presented in Table 1). There was a similar frequency of miscarriage in treated and untreated women; 12% (5/42) and 7.7% (3/39) respectively.

Although 81 women without miscarriage completed the study, chemokines and cytokines were not measured in four women because of missing samples (Fig. 1). Of the remaining 77 women, chemokine levels were compared between women with an early miscarriage (n = 7) and women with a pregnancy beyond gw 22 (n = 70). CXCL11 was the only chemokine that differed between groups (Student’s t-test on log-transformed data p = 0.013), showing significantly higher levels at inclusion, before treatment was started, in women who miscarried (geometric mean 49 pg/ml, 95% CI: 30–98) compared with the women who continued their pregnancy > gw 22 (geometric mean 27 pg/ml, 95% CI: 24–31, Supplementary Fig. 1).

### Longitudinal comparisons of cytokine and chemokine plasma levels in LMWH-treated versus non-treated women

**Figure 2:**
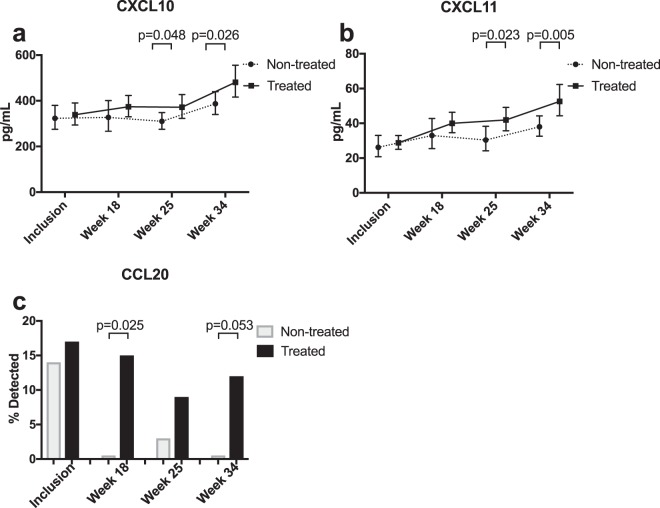
Chemokine levels in low-molecular-weight-heparin (LMWH) treated (n = 35) and non-treated (n = 35) women with unexplained pregnancy loss during pregnancy. (**a**,**b**) Longitudinal levels of CXCL10 (**a**) and CXCL11 (**b**) during pregnancy. Linear Mixed Models of log-transformed data showed differences between treated and untreated women; CXCL10, p = 0.007; CXCL11, p = 0.000. Student’s t-test (log-transformed data) was used as a post-hoc test to denote differences at specific time points. Geometric mean and 95% CI are shown (**c**). Linear Mixed Models on log-transformed data showed a difference in CCL20 levels between treated and untreated women during pregnancy (p = 0.037). Fisher’s exact test was used as a post-hoc test, proportion (%) of women with detectable levels are shown.

A mixed models test based on all samples during pregnancy (inclusion, gw 18, 28 and 34) showed that LMWH-treated women differed compared with non-treated women in plasma levels of the Th1-associated chemokines CXCL10 (p = 0.007) and CXCL11 (p = 0.000) (Fig. 2a,b, Table 3.) A subsequent unpaired Student’s t-test showed significantly higher levels of both these chemokines at gestational weeks 28 (CXCL10 p = 0.048, CXCL11 p = 0.023) and 34 (CXCL10 p = 0.026, CXCL11 p = 0.005) in treated compared with untreated women. The mixed models test also showed a significant difference (p = 0.037) between the treated and the untreated groups regarding the Th17-associated chemokine CCL20 (Fig. 2c). Since CCL20 levels were below the limit of detection in many samples, Fisher’s exact test was used for post hoc testing, showing more samples with detectable levels in treated women (15% and 12% at gw 18 and 34 respectively) compared with untreated women (0% at both gw 18 and 34, p = 0.025 and p = 0.053 respectively). Except for CXCL10, CXCL11 and CCL20, linear mixed models did not show any other differences in the cytokine and chemokine levels throughout pregnancy when comparing the treated and untreated groups (Table 3), and therefore no further post-hoc testing was done. With the exception of CCL20, the analysed cytokines and chemokines were present at detectable levels in most samples (Supplementary Table 2). Cytokine and chemokine levels 2 weeks’ *post partum* did not differ significantly between the treated and the untreated groups (data not shown).

**Table 3:** Cytokines/chemokines and their concentrations (pg/mL, geometric mean and 95% confidence interval) during pregnancy in the LMWH-treated group and the untreated control group.

Cytokine Chemokine	Old name	Associated response	Gestational week	LMWH geometric mean pg/mL (95% CI)		Control geometric mean pg/mL (95% CI)		p-value
CCL2	MCP-1	Th2	inclusion	150	(135–168)	156	(136–174)	0.278
			gw 18	162	(147–179)	163	(143–185)	0.827
			gw28	147	(131–165)	138	(123–151)	0.589
			gw34	148	(133–164)	142	(125–159)	0.606
CCL17	TARC	Th2	inclusion	9.5	(7.8–11)	10	(8.0–13)	0.341
			gw 18	8.3	(6.7–10)	8.2	(6.6–10)	0.990
			gw28	6.1	(4.8–7.8)	5.7	(4.6–7.2)	0.819
			gw34	5.4	(4.3–6.8)	5.0	(4.1–6.0)	0.691
**CCL20** ^a^	LARC	Th17	inclusion	6.0	(5.2–7.2)	5.5	(5.0–6.4)	0.565
			gw 18	**5.6** ^b^	**(5.0–6.4)**	**4.9**	**(4.9–4.9)**	**0.026**
			gw28	5.3	(4.9–5.8)	5.2	(4.9–6.2)	0.931
			gw34	**5.5** ^b^	**(5.0–6.3)**	**4.9**	**(4.9–4.9)**	**0.048**
CCL22	MDC	Th2	inclusion	639	(557–746)	555	(481–643)	0.282
			gw 18	549	(481–633)	480	(419–548)	0.187
			gw28	446	(387–519)	393	(338–455)	0.265
			gw34	430	(378–494)	409	(353–474)	0.567
CXCL1	GRO-α	Th17	inclusion	371	(282–471)	416	(332–530)	0.519
			gw 18	448	(345–574)	471	(372–610)	0.959
			gw28	400	(295–538)	373	(294–475)	0.724
			gw34	384	(282–508)	342	(274–436)	0.508
CXCL8	IL-8	Recruit neutrophil granulocytes	inclusion	8.6	(5.1–14)	12	(7.9–18)	0.085
			gw 18	7.7	(4.9–12)	11	(7.4–17)	0.115
			gw28	7.3	(4.6–12)	11	(6.8–17)	0.186
			gw34	7.9	(4.4–11)	11	(6.6–16)	0.155
**CXCL10** ^a^	IP-10	Th1	inclusion	349	(301–401)	317	(273–357)	0.656
			gw 18	379	(335–427)	323	(272–390)	0.134
			gw28	**377** ^c^	**(324–428)**	**310**	**(276–348)**	**0.048**
			gw34	**481** ^c^	**(413–551)**	**387**	**(336–443)**	**0.026**
**CXCL11** ^a^	IP-9 I-TAC	Th1	inclusion	28	(25–33)	25	(21–31)	0.485
			gw 18	41	(35–47)	33	(26–42)	0.126
			gw28	**42** ^c^	**(36–49)**	**30**	**(25–37)**	**0.023**
			gw34	**53** ^c^	**(45–62)**	**38**	**(33–45)**	**0.005**
CXCL12	SDF1	Th2 and recruit monocytes	inclusion	2065	(1845–2311)	2024	(1764–2326)	0.575
			gw 18	1841	(1626–2075)	1711	(1488–1975)	0.389
			gw28	1497	(1284–1757)	1358	(1108–1625)	0.542
			gw34	1496	(1250–1814)	1403	(1183–1660)	0.486
CXCL13	BLC, BCA-1	Recruit B-cells in inflammation	inclusion	131	(93–188)	134	(95–182)	0.730
			gw 18	216	(162–297)	204	(157–264)	0.829
			gw28	142	(105–195)	121	(94–154)	0.378
			gw34	151	(111–209)	132	(100–173)	0.600
IL-6	-	General inflammation	inclusion	9.6	(4.7–21)	14	(7.8–24)	0.470
			gw 18	8.6	(4.5–18)	12	(6.7–23)	0.401
			gw28	6.5	(3.4–14)	11	(5.6–21)	0.266
			gw34	6.4	(3.5–13)	13	(7.2–23)	0.152

MCP-1: Monocyte chemotactic protein, TARC: Thymus and activation-regulated chemokine, LARC: Liver and activation-regulated chemokine, MDC: Macrophage-derived chemokine, GRO-alpha: Growth-regulated alpha protein, IL: Interleukin, IP: Interferon gamma-inducible protein, I-TAC: Interferon-inducible T-cell alpha chemoattractant, SDF-1: Stromal cell-derived factor 1, BLC: B lymphocyte chemoattractant, BCA: B cell-attracting chemokine, pg: picogram, SD: Standard deviation.

^a^Linear mixed models show significant differences (in bold) between treated and untreated women based on all measured values during pregnancy; ^b^Fisher’s exact test shows significant difference between treated and untreated women at these time points ^c^Student’s t-test shows significant difference at these time points. For details see Results and Fig. 2.

### Descriptive data of obstetrical outcomes

The live birth rate was high in both the treated (86%) and the untreated (90%) groups (Table 2). The studied women with uRPL had a nominally higher rate of premature births, both in the study and the control group with 12% and 13%, respectively, compared with the background population in Sweden with a premature rate of 4% for live births^44^. The prevalence of intrauterine foetal deaths (IUFD) was nominally higher in the studied population (3%) compared with the background population where the incidence of IUFD is 3–4/1000^44^. The occurrence of preeclampsia, placental abruption, caesarean delivery or postpartum bleeding was similar in the treated and the untreated groups, as were neonatal parameters including mean gestational age (weeks), occurrence of small for gestational age and mean birthweight (Table 2).

### Adverse events

No serious maternal adverse events were reported. There were small vaginal bleedings in four treated women and three untreated women, and heavy bleeding at childbirth in two untreated women. None of the LMWH-treated women had heparin-induced thrombocytopaenia and only one woman was reported to have pain or bruising at injection sites, although this could be underreported from patients as this information was not systematically requested. Regarding foetal malformations, one pleural effusion was found in the treated group and one gastroschisis in the untreated group. Intrauterine growth restriction was seen in three cases with treatment and none in the untreated group, while neonatal death occurred in one of the treated women and one IUFD occurred in each group.

## Discussion

In this RCT of *in vivo* LMWH effects in pregnant women with uRPL, the main finding was a significant increase in plasma levels of the Th1-associated chemokines, CXCL10 and CXCL11, and possibly also of the Th17-associated chemokine, CCL20, in the second and third trimesters of pregnancy in women treated with LMWH compared to a control group without treatment. In contrast, LMWH did not decrease proinflammatory or increase anti-inflammatory/Th2-associated cytokines and chemokines. Thus, the only effects recorded would be supposedly unfavourable in relation to pregnancy, while other pregnancy-promoting effects not covered in the 11-plex panel may still occur.

To our knowledge, this is the first study assessing the immunological effects of LMWH *in vivo* in women with uRPL. Strengths in this study were the design as a randomised controlled study and the longitudinal sampling during the whole pregnancy. Also, we used a relevant panel of markers and not just one marker, and in general the analytes were measurable in plasma. A potential limitation was that peripheral blood does not represent the foetal–maternal interface environment. On the other hand, LMWH cannot pass over the placental barrier^45^ and can therefore directly modify only the maternal side during placentation and during pregnancy. As it was an open study, the women and the healthcare providers were aware of that the women did or did not get the treatment, which could have influenced psychological mechanisms that in turn might affect inflammation^46^. Participation in a study suggests possible “tender, love and care” effects, which could also have psychological effects in a positive direction^47,48^. The clinical outcome, which showed a similar rate of pregnancy complications in the treated and non-treated group, support the notion of a strong “tender, love and care” effect. This effect might also be described by the higher pregnancy success rate than expected, based on the historical success rate in a similar population^47,48^ The high pregnancy success rate could also be explained by the inclusion criteria that only viable pregnancies confirmed by ultrasound were included.

A consistent finding in our study was the increased plasma levels of the Th1-associated chemokines CXCL10 and CXCL11, which showed significantly higher levels in gestational weeks 28 and 34 in LMWH-treated compared with untreated women. CXCL10 and CXCL11 are both induced by IFN-γ and they bind to the same CXCR3 receptor on Th1 cells, which they both recruit^28,29,34^. Thus, their close relationship strengthens the biological relevance of the finding. A Th1 skewed immune response is known to affect pregnancy outcomes negatively in both murine models and in humans^8,23,25,43,49–53^. Thus, the Th1-associated and potentially proinflammatory effects of LMWH that we observed *in vivo* are rather contradictory to the hypothesised anti-inflammatory and pregnancy-promoting effects that have been assigned to LMWH treatment. A Th1 skewing by LMWH is in line with our previous *in vitro* findings where LMWH enhanced production of the Th1 associated cytokine IFN-ɣ^24^, the cytokine that induces production of CXCL10 and CXCL11. Although LMWH treatment was associated with increased plasma levels of CXCL10 and CXCL11, we cannot draw any conclusion from the present study regarding the clinical effect of this finding.

Of note, a controlled Th1-associated proinflammatory setting is important in the early implantation phase (and during the window of implantation)^25,54,55^. This setting leads to the expression of genes implicated in endometrial receptivity, which is later followed by an anti-inflammatory response essential for post-implantation embryo survival and development, and for a balanced trophoblast invasion^56^. The early potential requirement of a Th1 response could imply that LMWH should rather be used at an earlier time point during pregnancy.

Although the role of Th17 in pregnancy is uncertain, it is known to reflect a mainly proinflammatory profile^39^. Our study showed that the Th17-associated chemokine CCL20 at gw 18 and 34 was more often above the detection limit in the women treated with LMWH compared with untreated women. The low detectability of CCL20 complicates the interpretation; still it is likely to be higher in the treated group. Higher levels of Th17-associated CCL20 would be unfavourable considering the reported increase in Th17-associated responses in non-pregnant women with a history of uRPL^43^. Th17 is also relevant in the context of a ratio to Treg cells since these subsets show a plasticity to switch to one another and since Treg cells are main inducers of tolerance during pregnancy^39–42^. Our earlier *in vitro* study showed that LMWH decreased Treg cells and increased Th17-associated CCL20 production^24^. In the present study, we did not evaluate markers of Treg cells since they do not have a specific profile in chemokine receptor expression. A previous *in vivo* study of women with thrombophilia showed that LMWH-treated women had higher levels of Treg cells compared to women without LMWH treatment during pregnancy^57^. However, Foxp3 was the only marker used to define Treg cells, which implies a risk of including activated non-suppressive Foxp3-expressing cells^58,59^. Also, the scenario of lowered Treg cells in thrombophilia may not necessary be applied in the context of uRPL. Still, it would be very relevant to evaluate further any LMWH-mediated effects on Treg cells *in vivo*.

The lack of significant differences between treated and untreated women regarding all other analytes investigated (n = 8; IL-6, CCL2, CCL22, CCL17, CXCL1, CXCL8, CXCL12 and CXCL13) indicates that LMWH does not have a major impact on several specific circulating cytokines and chemokine during pregnancy. Notably, Lissauer *et al*.^43^ found Th1 and Th17-associated responses to be increased in non-pregnant women with a history of uRPL, *i.e*. the type of responses that we found to be increased by LMWH treatment *in vivo*.

Another finding of interest concerns the significantly higher plasma levels at inclusion (before treatment) of Th1-associated CXCL11 in women who later, regardless of treatment, miscarried compared to women who continued their pregnancy after gw 22. This finding is in line with several studies suggesting an enhanced systemic inflammatory response in women with uRPL, with higher levels of inflammatory cytokines, such as TNF, IFN-γ and IL-6, is a risk factor for miscarrying^49,50,60–64^. Although a controlled proinflammatory profile is necessary for proper implantation and development of pregnancy, a too strong inflammatory response may be detrimental^23,54,55^.

Women with uRPL are often treated with empirical medical treatments without scientific evidence^4^. It is thus of great importance to study whether any treatment can help this subgroup to have a better prognosis. This study was not powered to answer if LMWH can improve any clinical outcome in women with uRPL, instead the focus was to assess any immunological changes induced by LMWH. As we found immunological differences not in favour of using LMWH as an anti-inflammatory drug during the second and third trimesters of pregnancy, our data do not support further RCTs to evaluate clinical outcomes using LMWH during these gestational weeks. Further studies are needed to explore the hypothesis of using LMWH-associated proinflammatory effects to ameliorate the defective implantation process in RPL in the earliest stages of pregnancy.

## Conclusion

The findings indicate a potential proinflammatory effect of LMWH treatment *in vivo*. The observed Th1 and possibly also Th17-like deviations do not support a beneficial immune effect of LMWH *in vivo* during the second and third trimesters of pregnancy.

## Supplementary information

Dataset 1

## References

[1] de Jong PG, Kaandorp S, Di Nisio M, Goddijn M, Middeldorp S (2014). Aspirin and/or heparin for women with unexplained recurrent miscarriage with or without inherited thrombophilia. Cochrane Database Syst. Rev..

[2] The ESHRE Guideline Group on RPL. et al. (2018). ESHRE guideline: recurrent pregnancy loss. Hum. Reprod. Open.

[3] Atik, R. B. *et al*. ESHRE guideline: recurrent pregnancy loss. **10**, 1–12 (2018).

[4] Rasmark Roepke E (2018). Treatment efficacy for idiopathic recurrent pregnancy loss - a systematic review and meta-analyses. Acta Obs. Gynecol Scand.

[5] Gris J-CR (2011). LMWH have no place in recurrent pregnancy loss: debate-against the motion. Thromb. Res..

[6] Shaaban OM (2017). Low-Molecular-Weight Heparin for the Treatment of Unexplained Recurrent Miscarriage With Negative Antiphospholipid Antibodies: A Randomized Controlled Trial. Clin. Appl. Thromb..

[7] Monien S (2009). Use of Heparin in Women With Early and Late Miscarriages With and Without Thrombophilia. Clin. Appl. Thromb..

[8] Schleussner E (2015). Low-molecular-weight heparin for women with unexplained recurrent pregnancy loss: a multicenter trial with a minimization randomization scheme. Ann. Intern. Med..

[9] Pasquier E (2015). Enoxaparin for prevention of unexplained recurrent miscarriage: a multicenter randomized double-blind placebo-controlled trial. Blood.

[10] Clark P (2010). SPIN (Scottish Pregnancy Intervention) study: a multicenter, randomized controlled trial of low-molecular-weight heparin and low-dose aspirin in women with recurrent miscarriage. Blood.

[11] Kaandorp SP (2010). Aspirin plus heparin or aspirin alone in women with recurrent miscarriage. N Engl J Med.

[12] Hills FA (2006). Heparin prevents programmed cell death in human trophoblast. Mol. Hum. Reprod..

[13] Di Simone N (2007). Low-molecular weight heparin induces *in vitro* trophoblast invasiveness: role of matrix metalloproteinases and tissue inhibitors. Placenta.

[14] Tersigni C (2012). *In vitro* evidences of heparin’s effects on embryo implantation and trophoblast development. Reprod. Sci..

[15] Wang L, Brown JR, Varki A, Esko JD (2002). Heparin’s anti-inflammatory effects require glucosamine 6-O-sulfation and are mediated by blockade of L- and P-selectins. J. Clin. Invest..

[16] Zenerino C (2017). The HMGB1/RAGE pro-inflammatory axis in the human placenta: Modulating effect of low molecular weight heparin. Molecules.

[17] Park H (2005). A distinct lineage of CD4 T cells regulates tissue inflammation by producing interleukin 17. Nat. Immunol..

[18] Yan Y (2017). Non-anticoagulant effects of low molecular weight heparins in inflammatory disorders: A review. Carbohydr. Polym..

[19] Mousavi, S., Moradi, M., Khorshidahmad, T. & Motamedi, M. Anti-inflammatory effects of heparin and its derivatives: A systematic review. *Adv. Pharmacol. Sci*. 2015, (2015). 10.1155/2015/507151 PMC4443644 26064103

[20] Attanasio M (1998). Cytokine gene expression in human LPS- and IFNgamma-stimulated mononuclear cells is inhibited by heparin. Thromb. Haemost..

[21] Høgåsen AK, Abrahamsen TG (1995). Heparin suppresses lipopolysaccharide-induced monocyte production of several cytokines, but simultaneously stimulates C3 production. Thromb. Res..

[22] Poterucha TJ, Libby P, Goldhaber SZ (2017). More than an anticoagulant: Do heparins have direct anti-inflammatory effects?. Thromb. Haemost..

[23] Christiansen OB (2013). Reproductive immunology. Mol. Immunol..

[24] Bruno V (2018). Effects of low molecular weight heparin on the polarization and cytokine profile of macrophages and T helper cells *in vitro*. Sci. Rep..

[25] Mor G, Aldo P, Alvero AB (2017). The unique immunological and microbial aspects of pregnancy. Nat Rev Immunol.

[26] Abrahamsson TR, Sandberg Abelius M, Forsberg A, Björkstén B, Jenmalm MC (2011). A Th1/Th2-associated chemokine imbalance during infancy in children developing eczema, wheeze and sensitization. Clin. Exp. Allergy.

[27] Henningsson AJ, Tjernberg I, Malmvall BE, Forsberg P, Ernerudh J (2011). Indications of Th1 and Th17 responses in cerebrospinal fluid from patients with Lyme neuroborreliosis: A large retrospective study. J. Neuroinflammation.

[28] Griffith JW, Sokol CL, Luster AD (2014). Chemokines and chemokine receptors: positioning cells for host defense and immunity. Annu Rev Immunol.

[29] Ekman AK (2013). Systemically elevated Th1-, Th2- and Th17-associated chemokines in psoriasis vulgaris before and after ultraviolet B treatment. Acta Derm. Venereol..

[30] Annunziato F, Cosmi L, Liotta F, Maggi E, Romagnani S (2012). Defining the human T helper 17 cell phenotype. Trends Immunol..

[31] Mantovani A (2004). The chemokine system in diverse forms of macrophage activation and polarization. Trends Immunol..

[32] Hayglass KT (2011). The quest for predictive immune biomarkers. Clin. Exp. Allergy.

[33] Zhu J, Paul WE (2008). CD4 T cells: fates, functions, and faults. Blood.

[34] Qin S (1998). The chemokine receptors CXCR3 and CCR5 mark subsets of T cells associated with certain inflammatory reactions. J. Clin. Invest..

[35] Islam SA, Luster AD (2012). T cell homing to epithelial barriers in allergic disease. Nat. Med..

[36] Piao HL (2012). The CXCL12/CXCR4 axis is involved in the maintenance of Th2 bias at the maternal/fetal interface in early human pregnancy. Cell. Mol. Immunol..

[37] Joseph PRB, Sawant KV, Rajarathnam K (2017). Heparin-bound chemokine CXCL8 monomer and dimer are impaired for CXCR1 and CXCR2 activation: implications for gradients and neutrophil trafficking. Open Biol..

[38] Svensson J (2011). Macrophages at the Fetal-Maternal Interface Express Markers of Alternative Activation and Are Induced by M-CSF and IL-10. J. Immunol..

[39] Saito S, Nakashima A, Shima T, Ito M (2010). Th1/Th2/Th17 and Regulatory T-Cell Paradigm in Pregnancy. Am. J. Reprod. Immunol..

[40] Ernerudh J, Berg G, Mjösberg J (2011). Regulatory T helper cells in pregnancy and their roles in systemic versus local immune tolerance. Am. J. Reprod. Immunol..

[41] Figueiredo AS, Schumacher A (2016). The T helper type 17/regulatory T cell paradigm in pregnancy. Immunology.

[42] Robertson SA, Care AS, Moldenhauer LM (2018). Regulatory T cells in embryo implantation and the immune response to pregnancy The. Journal of Clinical Investigation. J Clin Invest.

[43] Lissauer D, Goodyear O, Khanum R, Moss PAH, Kilby MD (2014). Profile of maternal CD4 T-cell effector function during normal pregnancy and in women with a history of recurrent miscarriage. Clin. Sci. (Lond)..

[44] The National Board of Health and Welfare. No Title. Swedish Official Statistics. Pregnancies, deliveries and newborn babies., https://www.socialstyrelsen.se/Lists/Artikelkatalog/Attachments/20009/2015-12=27.p (2015).

[45] Andrew M (1985). Placental transport of low molecular weight heparin in the pregnant sheep. Br. J. Haematol..

[46] Arck PC (2001). Stress and immune mediators in miscarriage. Hum. Reprod..

[47] Stray-Pedersen B, Stray-Pedersen S (1984). Etiologic factors and subsequent reproductive performance in 195 couples with a prior history of habitual abortion. Am. J. Obstet. Gynecol..

[48] Clifford K, Rai R, Regan L (1997). Future pregnancy outcome in unexplained recurrent first trimester miscarriage. Hum Reprod.

[49] Jenkins C (2000). Evidence of a T(H) 1 type response associated with recurrent miscarriage. Fertil. Steril..

[50] Raghupathy R (1999). Maternal Th1- and Th2-type reactivity to placental antigens in normal human pregnancy and unexplained recurrent spontaneous abortions. Cell. Immunol..

[51] Svensson-Arvelund J (2015). The human fetal placenta promotes tolerance against the semiallogeneic fetus by inducing regulatory T cells and homeostatic M2 macrophages. J. Immunol..

[52] Pomin VH, Mulloy B (2015). Current structural biology of the heparin interactome. Curr. Opin. Struct. Biol..

[53] Boij R (2012). Biomarkers of Coagulation, Inflammation, and Angiogenesis are Independently Associated with Preeclampsia. Am. J. Reprod. Immunol..

[54] Szarka A, Rigo J, Lazar L, Beko G, Molvarec A (2010). Circulating cytokines, chemokines and adhesion molecules in normal pregnancy and preeclampsia determined by multiplex suspension array. BMC Immunol.

[55] Robertson SA, Chin PY, Femia JG, Brown HM (2018). Embryotoxic cytokines—Potential roles in embryo loss and fetal programming. J. Reprod. Immunol..

[56] Salker MS (2012). Disordered IL-33/ST2 activation in decidualizing stromal cells prolongs uterine receptivity in women with recurrent pregnancy loss. PLoS One.

[57] Luley L (2015). Low molecular weight heparin modulates maternal immune response in pregnant women and mice with thrombophilia. Am. J. Reprod. Immunol..

[58] Mjösberg J, Berg G, Jenmalm MC, Ernerudh J (2010). FOXP3+ Regulatory T Cells and T Helper 1, T Helper 2, and T Helper 17 Cells in Human Early Pregnancy Decidua1. Biol. Reprod..

[59] Miyara M (2009). Functional Delineation and Differentiation Dynamics of Human CD4+T Cells Expressing the FoxP3 Transcription Factor. Immunity.

[60] Raghupathy R (2000). Cytokine production by maternal lymphocytes during normal human pregnancy and in unexplained recurrent spontaneous abortion. Hum. Reprod..

[61] Hill J, Polgar K, Anderson D (1995). T-helper 1-type immunity to trophoblast in women with recurrent spontaneous abortion. Jama.

[62] Sehmsdorf US (2004). Human miscarriage is associated with increased number of CD26+ decidual lymphocytes. Scand. J. Immunol..

[63] Makhseed M (2001). Th1 and Th2 cytokine profiles in recurrent aborters with successful pregnancy and with subsequent abortions. Hum. Reprod..

[64] Zenclussen AC (2001). Upregulation of decidual P-Selectin expression is associated with an increased number of Th1 cell populations in patients suffering from spontaneous abortions. Cell. Immunol..

