# Author Correction: Low-molecular-weight-heparin increases Th1- and Th17-associated chemokine levels during pregnancy in women with unexplained recurrent pregnancy loss: a randomised controlled trial

**DOI:** 10.1038/s41598-020-67807-8

**Published:** 2020-06-25

**Authors:** E. Rasmark Roepke, V. Bruno, E. Nedstrand, R. Boij, C. Petersson Strid, E. Piccione, G. Berg, J. Svensson-Arvelund, M. C. Jenmalm, M. Rubér, J. Ernerudh

**Affiliations:** 1 Department of Obstetrics and Gynecology, Skåne University Hospital, Malmö and Lund University, Lund, Sweden; 2 Department of Clinical and Experimental Medicine, Linköping University, Linköping, Sweden; 3 Departmen of Obstetrics and Gynecology, Kalmar Hospital, Kalmar, Sweden; 4 Section of Gynecology and Obstetrics, Academic Department of Biomedicine and Prevention, and Clinical Department of Surgery, Tor Vergata University Hospital, Rome, Italy; 5 Department of Clinical Immunology and Transfusion Medicine, and Department of Clinical and Experimental Medicine, Linköping University, Linköping, Sweden

Correction to: *Scientific Reports* 10.1038/s41598-019-48799-6, published online, 23 August 2019

This Article contained an error in the Author Information section where:

“E. Rasmark Roepke, V. Bruno, M. Rubér and J. Ernerudh contributed equally.”

now reads:

“E. Rasmark Roepke and V. Bruno contributed equally. M. Rubér and J. Ernerudh jointly supervised this work.”

This error has now been corrected in the PDF and HTML versions of the Article.

