# Supplementary material for: Low-molecular-weight-heparin increases Th1- and Th17-associated chemokine levels during pregnancy in women with unexplained recurrent pregnancy loss: a randomised controlled trial
Source: Sci Rep. 2019 Aug 23;9:12314. doi: 10.1038/s41598-019-48799-6 (PMC6707182; doi:10.1038/s41598-019-48799-6)
Supplement: Supplementary file 1 — Dataset 1 [file 41598_2019_48799_MOESM1_ESM.docx]

**Low-molecular-weight-heparin increases Th1- and Th17-associated chemokine levels during pregnancy in women with unexplained recurrent pregnancy loss, a randomised controlled trial.**

^Ϯ*^E Rasmark Roepke1,^Ϯ^V Bruno^2,4^, E Nedstrand^2^, R Boij^2^, C Petersson Strid^3^, E Piccione^4^, G Berg^2^, J Svensson-Arvelund^2^, M.C Jenmalm^2^, ^#^M Rubér^2^, ^#^J Ernerudh^5^

^Ϯ #^ These authors contributed equally
* Corresponding author

Supplementary Table S1. Inclusion and exclusion criteria

| Inclusion criteria | Exclusion criteria | Withdrawal criteria |
| --- | --- | --- |
| RPL; ≥ 3 consecutive miscarriages | Ongoing treatment for RPL other than LMWH | Severe adverse event not connected to pregnancy complication |
| Unknown aetiology to RPL | Known aetiology to RPL | Severe side effects connected to tinzaparin (thrombocytopaenia, severe bleeding, eczema rash) |
| Age ≥18 to ≤42 years old | Antiphopholipidsyndrome  Thrombophilia  Ongoing infection  Diabetes mellitus type 1 and 2  Poorly regulated thyroid disease  Autoimmune disease  Kidney insufficiency  Haemorrhagic diathesis  Allergic to tinzaparin  Heparin associated thrombocytopaenia  Acute bacterial endocarditis  Injury or operation in the central nervous system in the last year  Participation in other studies, current or recently (30 days)  Smoking^[[1]](#footnote-1)^ | Simultaneous treatment with other anticoagulant |
| Informed consent from the patient |  | Protocol was not followed |

Supplementary Table S2. Numbers and proprtions of detectable values for cytokines and chemokines

| Cytokine/chemokine (lowest detectable value, pg/ml) | LMWH  n/total (%) | Control  n/total (%) | Total  n/total (%) |
| --- | --- | --- | --- |
| CCL2 (16)  inclusion  gw 18  gw 25  gw 35  2 w pp | 35/35 (100)  34/34 (100)  34/34 (100)  33/33 (100) 33/33 (100) | 35/35 (100)  35/35 (100)  33/33 (100) 34/34 (100)  35/35 (100) | 70/70 (100)  69/69 (100)  67/67 (100)  67/67 (100)  68/68 (100) |
| CCL17 (1.0)  inclusion  gw 18  gw 25  gw 35  2 w pp | 35/35 (100)  34/34 (100)  34/34 (100)  34/34 (100)  33/33 (100) | 35/35 (100)  35/35 (100)  33/33 (100)  33/33 (100)  34/35 (97) | 70/70 (100)  69/69 (100)  67/67 (100)  67/67 (100)  67/68 (99) |
| CCL20 (9.8)  inclusion  gw 18  gw 25  gw 35  2 w pp | 6/35 (17) 5/34 (15) 3/34 (9) 4/33 (12) 12/33 (36) | 5/35 (14) 0/35 (0) 1/33 (3) 0/34 (0) 7/35 (20) | 59/70 (84) 5/69 (7) 4/67 (6) 4/67 (6) 19/68 (28) |
| CCL22 (16)  inclusion  gw 18  gw 25  gw 35  2 w pp | 35/35 (100) 34/34 (100) 34/34 (100)  33/33 (100)  33/33 (100) | 35/35 (100) 35/35 (100)  33/33 (100) 34/34 (100) 35/35 (100) | 70/70 (100) 69/69 (100)  67/67 (100)  67/67 (100)  68/68 (100) |
| CXCL1 (16)  inclusion  gw 18  gw 25  gw 35  2 w pp | 35/35 (100) 34/34 (100) 34/34 (100) 33/33 (100) 33/33 (100) | 35/35 (100) 35/35 (100) 33/33 (100) 34/34 (100) 35/35 (100) | 70/70 (100) 69/69 (100) 67/67 (100) 67/67 (100)  68/68 (100) |
| CXCL8 (1.6)  inclusion  gw 18  gw 25  gw 35  2 w pp | 28/35 (80)  29/34 (85)  28/34 (82)  27/33 (82)  32/33 (97) | 33/35 (94)  34/35 (97)  31/33 (94)  30/34 (88)  34/35 (97) | 61/70 (87)  63/69 (91)  59/67 (88)  57/67 (85)  66/68 (97) |
| CXCL10 (16)  inclusion  gw 18  gw 25  gw 35  2 w pp | 35/35 (100)  34/34 (100) 34/34 (100)  33/33 (100)  33/33 (100) | 35/35 (100) 35/35 (100)  33/33 (100)  34/34 (100)  35/35 (100) | 70/70 (100)  69/69 (100)  67/67 (100)  67/67 (100)  68/68 (100) |
| CXCL11 (7.8)  inclusion  gw 18  gw 25  gw 35  2 w pp^[[2]](#footnote-2)^ | 35/35 (100)  34/34 (100)  33/33 (100)  33/33 (100)  33/33 (100) | 35/35 (100)  35/35 (100)  34/34 (100)  34/34 (100)  34/35 (97) | 70/70 (100)  69/69 (100)  67/67 (100)  67/67 (100)  67/68 (99) |
| CXCL12 (3.9)  inclusion  gw 18  gw 25  gw 35  2 w pp | 35/35 (100)  34/34 (100)  34/34 (100)  33/33 (100)  33/33 (100) | 35/35 (100)  35/35 (100)  32/33 (97)  34/34 (100)  35/35 (100) | 70/70 (100)  69/69 (100)  66/67 (99)  67/67 (100)  68/68 (100) |
| CXCL13 (3.9)  inclusion  gw 18  gw 25  gw 35  2 w pp | 35/35 (100)  34/34 (100)  34/34 (100)  33/33 (100)  33/33 (100) | 35/35 (100)  35/35 (100)  33/33 (100)  34/34 (100)  35/35 (100) | 70/70 (100)  69/69 (100)  67/67 (100)  67/67 (100)  68/68 (100) |
| IL6 (1.6) inclusion  gw 18  gw 25  gw 35  2 w pp | 23/35 (66) 24/34 (71) 21/34 (62)  21/33 (64) 28/33 (85) | 28/35 (80) 28/35 (80) 24/33 (73)  27/34 (79)  31/35 (89) | 51/70 (73) 52/69 (75) 45/67 (67) 48/67 (72)  59/68 (87)^[[3]](#footnote-3)^ |

Supplementary Figure S1.

**
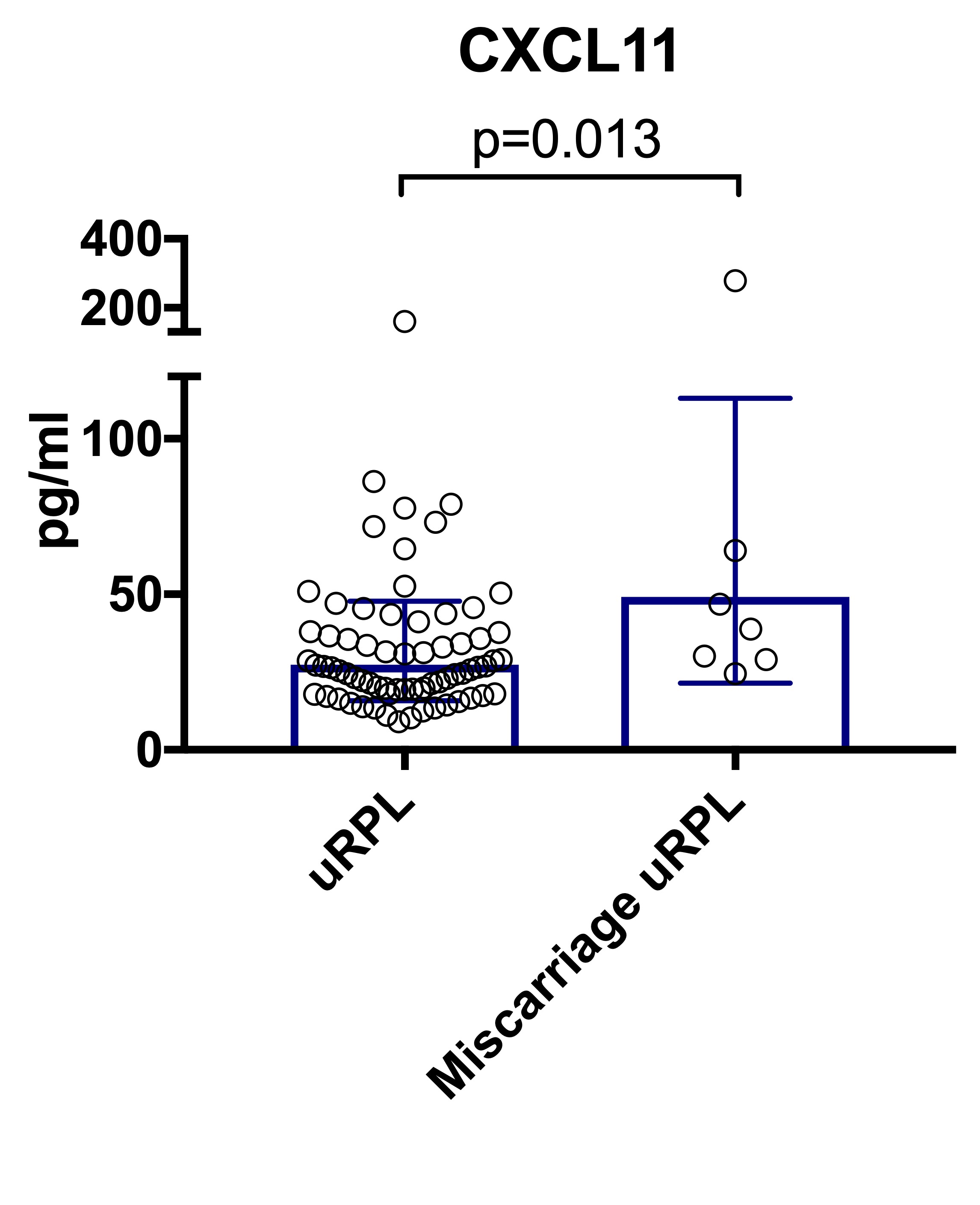
**

Supplementary Figure S1. CXCL11 levels at inclusion time in women who miscarried <gw22 (n=7; Miscarriage uRPL) and uRPL women who continued their pregnancy beyond gw 22 (n=70; uRPL). Individual values are shown and geometric mean and 95% CI are shown, Student´s t-test on log-transformed data.

1. RPL; recurrent pregnancy loss

   LMWH: low-molecular-weight heparin [↑](#footnote-ref-1)
2. [↑](#footnote-ref-2)
3. pg: picogram, gw: gestational week, w: week, pp: post partum

   Supplementary Table S3. Pregnancy outcome for women with complete blood samples

   | Pregnancy outcome | LMWH n=39  n (%) | Control n=38  n (%) |
   | --- | --- | --- |
   | Live birth rate | 34 (87) | 34 (90) |
   | Miscarriage | 4 (10) | 3 (7.9) |
   | Full-term labour | 30 (77) | 30 (80) |
   | Mode of delivery  Partus normal vaginal  Cesearean, elective/acute  Vacuum or forceps | 20 (51)  10 (26), 4/6  5 (13) | 27 (71)  5 (13), 1/4  3 (7.9) |
   | Obstetric complications  Preterm labour  Preeclampsia^b^  SGA^b^  Gestational diabetes  Bleeding post-partum > 1L  Placenta abruption  IUFD  Foetal death | 5^a^ (13)  2 (5.7)  3 (8.6)  0  0  1 (2.9)  1 (2.9)  0 | 5 ^a^ (13)  1 (2.9)  0  1 (2.9)  2 (5.7)  0  1 (2.9)  1 (2.9) |

   LMWH; low-molecular-weight heparin, gw; gestational week, SGA; small for gestational age, IUFD: intrauterine foetal death ^a^Includes IUFD
   ^b^Women with preeclampsia and SGA are not the same women.

   *Excluded women and women with missing blood samples are not included in the table [↑](#footnote-ref-3)
